# Ulnar fascicle to brachialis branch of musculocutaneous nerve for restoration of elbow flexion associated with spinal cord tumor and radiation-induced lower motor neuron disease

**DOI:** 10.3171/2022.10.FOCVID2299

**Published:** 2023-01-01

**Authors:** M. Benjamin Larkin, Eric A. Goethe, Mehreen Mohammad, Sudhakar Tummala, Robert Y. North

**Affiliations:** 1 Department of Neurosurgery, Baylor College of Medicine, Houston;; Departments of 2 Neurosurgery and; 3 Neuro-Oncology, The University of Texas MD Anderson Cancer Center, Houston, Texas

**Keywords:** nerve transfer, ulnar fascicle to biceps motor branch, radiation-induced lower motor neuron disease, Oberlin transfer

## Abstract

Transfer of the ulnar fascicle to the biceps branch of the musculocutaneous nerve, or Oberlin transfer, has been widely used for the treatment of elbow flexion weakness in the setting of upper trunk brachial plexus palsy. The authors present a modified application of this technique for restoration of functional elbow flexion in a 30-year-old woman with a history of recurrent upper cervical spinal cord pilocytic astrocytoma, complex spinal deformity, and radiation-induced lower motor neuron disease.

The video can be found here: https://stream.cadmore.media/r10.3171/2022.10.FOCVID2299

## Transcript

This video illustrates the application of an ulnar nerve fascicle to the brachialis branch of the musculocutaneous nerve, for the restoration of elbow flexion due to weakness, related to radiation-induced motor neuron disease, and a recurrent upper cervical spinal cord pilocytic astrocytoma, as well as an associated complex spinal column deformity.

### 0:41 History of Present Illness.

The patient was a 30-year-old female, with more than a 20-year history of an upper cervical spinal cord pilocytic astrocytoma. She had undergone several surgical resections, radiation treatments, and a syrinx fenestration. She also previously underwent an anterior and posterior cervical spine fusion to correct her kyphotic deformity, and a thoracolumbar fusion for neuromuscular associated scoliosis. The patient presented for an opinion on the management of residual tumor throughout her cervicomedullary junction that continued inferiorly to the level of C3.

Since 2007, the patient has had longstanding weakness in her left arm and spastic paresis in her bilateral lower extremities. These limitations made the patient completely reliant on her right upper extremity for performing work tasks and other activities of daily living. Now, she presents to clinic and reports a progressive right upper-extremity weakness that developed 5 years prior with shoulder weakness, has now worsened over the past year, and now includes severe elbow flexion weakness. This has left her no longer able to feed herself or carry out many activities of daily living independently.

Recommendations from multiple centers, including our own, were that further surgical resection of the tumor or additional correction of the spinal deformity offered unclear benefits with an exceptionally high risk of complications.

### 2:13 Physical Examination

On exam, she had significant weakness in both arms but had retained the strength in her right hand. She had no discernable upper-extremity reflexes. More specifically, in her right arm and hand, she had only MRC grade 1–2 strength for shoulder abduction and elbow flexion but retained at least MRC grade 4 for grip strength and finger abduction.

Clinical examination, EMG, and nerve conduction study were consistent with radiation-induced lower motor neuron disease superimposed on cervical myelopathy.

While peripheral nerve and brachial plexus injuries are considered the most common indications for nerve transfer surgeries, it has also been applied to other neurological disorders, which cause significant morbidity and functional limitations, including patients with a history of stroke or spinal cord injuries.^1–3^ While it was felt that this patient was not a candidate for further tumor resection, she was offered a nerve transfer in the hope of restoring elbow flexion strength given the significant functional limitations she now faced with her progressive right arm weakness.

### 3:23 Background.

The ulnar fascicle to biceps branch of the musculocutaneous nerve, also known as the Oberlin nerve transfer, was originally described in 1994 by Oberlin et al. for the treatment of C5 to C6 brachial plexus avulsion injuries.^4^ In their original series, three of the four patients regained at least MRC grade 4 elbow flexion. Other authors, to supplement this transfer, have described the additional transfer of a median nerve fascicle to brachialis branch of the musculocutaneous nerve.^5^ In this case, however, the brachialis muscle was selected as the recipient for the ulnar fascicle donor. This was because when tested preoperatively, the patient’s biceps strength had a stronger palpable contraction compared to the brachialis, as well as the patient’s relative preservation of supination. Thus, we felt that the restoration of the patient’s brachialis muscle strength would yield the greatest benefit to elbow flexion.

### 4:22 Surgery.

The donor and recipient nerves are found within the medial upper arm. A straight linear skin incision is marked in the interval between the triceps and the biceps muscles, extending along the anterior axillary line. The incision is then carefully deepened through the subcutaneous tissue. Typically, the first nerve encountered is the medial antebrachial cutaneous nerve, and this serves as an important landmark. The next structure that should be identified is the basilic vein and is encountered immediately deep to the medial antebrachial cutaneous nerve. A deepened dissection, just medial to the basilic vein, will often lead to the ulnar nerve. After the isolation and looping of the ulnar nerve, attention can then be turned toward the identification and isolation of the musculocutaneous nerve and its branches. Once the biceps muscle belly is identified, it is then retracted in order to look for the musculocutaneous nerve, which lies on its inferior surface just above the brachialis muscle. During this portion, it is important to carefully dissect and protect the small branches innervating the biceps and brachialis that run in between these muscles. Once the recipient branch to the biceps or brachialis is identified, it is dissected as far proximally as possible, and then sharply transected from its musculocutaneous nerve origin. Next, the ulnar nerve is dissected circumferentially. During the intrafascicular portion of the dissection, intraoperative neuromonitoring of flexor carpi ulnaris, the first dorsal interosseous muscle, adductor pollicis, and abductor digiti minimi is utilized. The epineurium of the ulnar nerve is then opened with the use of an operative microscope, and then the nerve is carefully dissected until a fascicle of similar diameter to the recipient branch is identified. Afterward, selective stimulation of the isolated fascicle is used to confirm motor stimulation via triggered EMG within the monitored muscles. Prior to transection, stimulation of the remaining nerve is performed to confirm that the ulnar innervated muscles of the hand are represented.^6^

Before it is sharply divided, the selected donor fascicle is dissected as far distally as is possible, and proximally to the point perpendicular to the isolated recipient branch. The adjacent ends then are approximated using interrupted 9-0 nylon sutures placed through the epineurium, such that the suture line remains even and not buckled at any point. This helps to ensure that there is no tension throughout the coaptation. Afterward, the repair is covered with Tisseel fibrin sealant, and the incision closed in a standard fashion.

### 7:12 Conclusion.

We present a unique application of a peripheral nerve transfer, utilizing an ulnar nerve fascicle to brachialis branch transfer for the restoration of elbow flexion, in a patient with a lower motor neuron disease, secondary to a long history of a recurrent cervical spinal cord pilocytic astrocytoma and a complex spinal deformity.

### 7:33 Patient Outcome.

The patient did well and was discharged home on postoperative day 1, with an unchanged neurological exam. Six months postoperatively, she had regained antigravity elbow flexion. At her 10-month clinic follow-up, shown here, she had obtained 4 out of 5 elbow flexion strength and was once again able to eat and drink independently, as well as having improved function with many of her own activities of daily living.

Thank you for watching!

## Disclosures

The authors report no conflict of interest concerning the materials or methods used in this study or the findings specified in this publication.

## Author Contributions

Primary surgeon: North. Editing and drafting the video and abstract: North, Larkin, Goethe, Mohammad. Critically revising the work: North, Larkin, Goethe, Tummala. Reviewed submitted version of the work: North, Larkin, Goethe, Mohammad. Approved the final version of the work on behalf of all authors: North. Performed EMG and intraoperative examination and clinical neurology assessment: Tummala.
